# Microstructure-Based Flow Stress Model to Predict Machinability of Inconel 718

**DOI:** 10.3390/ma17174206

**Published:** 2024-08-25

**Authors:** Qingan Yin, Hui Chen, Jianxiong Chen, Yu Xie, Ming Shen, Yuhua Huang

**Affiliations:** 1 School of Mechanical Engineering and Automation, Fuzhou University, Fuzhou 350108, China; yqa@fzu.edu.cn (Q.Y.); felixceng@fzu.edu.cn (H.C.); jxchen045@fzu.edu.cn (J.C.); huangyuhua@fzu.edu.cn (Y.H.); 2 School of Mathematics and Statistics, Fuzhou University, Fuzhou 350108, China; shenming0516@fzu.edu.cn

**Keywords:** microstructure, flow stress model, machinability, Inconel 718

## Abstract

Due to its exceptional mechanical and chemical properties at high temperatures, Inconel 718 is extensively utilized in industries such as aerospace, aviation, and marine. Investigating the flow behavior of Inconel 718 under high strain rates and high temperatures is vital for comprehending the dynamic characteristics of the material in manufacturing processes. This paper introduces a physics-based constitutive model that accounts for dislocation motion and its density evolution, capable of simulating the plastic behavior of Inconel 718 during large strain deformations caused by machining processes. Utilizing a microstructure-based flow stress model, the machinability of Inconel 718 in terms of cutting forces and temperatures is quantitatively predicted and compared with results from orthogonal cutting experiments. The model’s predictive precision, with a margin of error between 5 and 8%, ensures reliable consistency and enhances our comprehension of the high-speed machining dynamics of Inconel 718 components.

## 1. Introduction

Nickel-based superalloys, renowned for their fatigue resistance, radiation tolerance, oxidation resistance, impact strength, and corrosion resistance, as well as their favorable processing characteristics, are extensively utilized in critical industries such as aerospace, nuclear energy, and petroleum. These alloys retain their mechanical and chemical properties even at elevated temperatures [1]. Among them, Inconel 718 stands out as a prevalent and extensively studied high-temperature nickel-based alloy, demonstrating a superior performance across a broad temperature spectrum ranging from −253 to 650 °C [2]. Its exceptional physical and chemical properties at high temperatures have led to its broad application in various demanding environments [3].

Materials undergo various loading conditions during processing, characterized by diverse strain rates and temperatures. The production of metallic components frequently entails deformation under high strain rates and temperatures, which is particularly evident in forming and machining operations [4,5]. Investigating the flow behavior of Inconel 718 under such conditions is crucial for comprehending its dynamic properties during manufacturing [6].

Material flow behavior under diverse loading conditions is typically described by constitutive models that account for the influences of strain, strain rate, and temperature [7]. Consequently, substantial research has been dedicated to developing and refining these models.

Xi et al. [8] indicated that the classical Johnson–Cook (JC) model falls short in describing the flow stress of Inconel 718 across different deformation temperatures and strain rates. Consequently, an enhanced JC constitutive model was developed, incorporating the coupled effects of temperature and strain rate, and it was used to forecast the flow stress curves at various temperatures. Del Prete et al. [9] proposed a modified material model considering the initial hardness values of the material’s influence on the JC constitutive model. With this model, it is feasible to simulate the machining process of Inconel 718 across varying hardness levels under the unified JC constitutive model.

Silva et al. [10], considering the mechanical behavior of Inconel 718 during machining, which includes its strain rate and stress state, proposed a constitutive model that characterizes the mechanical behavior of Inconel 718 in the machining process. Further predictions were made regarding the cutting forces, chip geometry, cutting temperature, and residual stress. A comparison of the predicted outcomes with actual measurements demonstrated that the orthogonal cutting model could adequately represent the machining process of Inconel 718. In 2014, Jafarian et al. [11] compared different material models from the literature with machining experimental results and concluded that the JC constitutive model is the most suitable for simulating Inconel 718 machining.

Among these models, the JC constitutive model is the most prevalent for use in machining simulations. The simplicity of the classical thermal–viscoplastic JC constitutive model, along with the universal applicability of its parameters for various metals, has led to its extensive use. This model characterizes the relationship between true stress and true strain under different deformation mechanisms at various strain rates and temperatures. The JC constitutive model is the most frequently utilized material model in the simulation of cutting processes, assuming material isotropy and that material strength is a function of strain, strain rate, and temperature [12]. Moreover, the adaptability of the JC constitutive model is bolstered by its compatibility with microstructure-dependent semiempirical models, facilitating the acquisition of microscale material modification insights. Conversely, the model often requires recalibration to accommodate diverse operational conditions, and the numerical constants derived often lack clear physical interpretations [13].

Simultaneously, the establishment of empirical constitutive models necessitates a substantial amount of experimentation. Consequently, researchers have conducted a series of studies on physical-based constitutive models. Denguir et al. [14] integrated the effects of stress state and microstructure on the material behavior of a workpiece, establishing a physical constitutive model for predicting the surface integrity of OFHC copper based on dislocation density. Compared to the classical Johnson–Cook model, the proposed constitutive model yielded superior predictive results.

On the basis of regression analysis, Pauskar and Shivpuri [15] proposed a microstructure-informed flow stress model. This flow stress model characterizes material behavior as a function of microscale phenomena, encompassing both strengthening effects—like dislocation interactions—and softening mechanisms—such as dynamic recovery, grain recrystallization, and grain boundary sliding. In this model, it is assumed that dynamic recovery is the only softening mechanism in the deformation process.

Estrin et al. [16] developed a physical model that forecasts the microstructural evolution occurring during forming processes by correlating flow stress with dislocation density. In this model, dislocation density is categorized into two distinct types: internal dislocations and wall dislocations. Two different dislocation evolutions are considered in the process of material deformation, i.e., the low-dislocation-density channel and the high-dislocation-density channel. Ding et al. [17] formulated a dislocation-density-based material model to simulate the grain refinement and dislocation behavior in Al 6061T6 and OFHC-Cu under varying cutting conditions.

Ding et al. [18] established a multi-physics model of the surface structure evolution and surface hardness changes occurring during the machining of AISI 52,100 steel. The multi-physics model was utilized to forecast microstructural alterations by considering transformation and grain refinement at the same time. Liu et al. [19] established a physical model considering the hardening and recovery effects of dislocations in the process of plastic deformation and the evolution of grain size, which had a good prediction effect. Fisk et al. [20] established a material model of Inconel 718 by considering the interaction between precipitates and dislocations based on the dislocation density.

Rotella and Umbrello [21] established a constitutive model to forecast the microstructural alterations (grain size and microhardness) of Ti6Al4V at a low temperature and during dry cutting by considering the influence of microstructure evolution (grain size) on flow stress. Atmani et al. [22] employed a physical model, the mechanical threshold stress model, and the JC model to characterize material behavior under thermoviscoplastic conditions. By integrating the physical dislocation density model with the mechanical threshold stress model, changes in the material microstructure during the cutting process were described.

Lindgren et al. [23] introduced a dislocation-density-based model that posits flow stress as an aggregate of various contributions. Its key components include the interaction of moving and immobile dislocations, microstructural alterations due to grain size variations, and the dynamics between moving dislocations and short-range obstacles. Zhu et al. [15] developed a comprehensive constitutive model incorporating grain boundary strengthening, precipitation strengthening, and solution strengthening to predict the flow stress behavior of nickel-based superalloys. The microstructural evolution of Inconel 718 was examined using optical microscopy (OM), electron backscatter diffraction (EBSD), and transmission electron microscopy (TEM).

Imbrogno et al. [24] formulated a physical-based constitutive model aimed at forecasting the surface integrity of machined Waspaloy, a nickel-based superalloy. This model posits that the overall macroscopic flow stress is an amalgamation of long-range and short-range contributions. Bacca et al. [25] introduced an innovative computational model that captures the grain size evolution occurring during intense plastic deformation, applying it to finite element analyses of machining for an Al-6061-T6 alloy. Nonetheless, this model’s applicability is confined to scenarios involving significant plastic deformation, where annealing effects are minimal.

Several studies have underscored the constraints of the JC constitutive model, notably, its omission of microstructural effects on the mechanical properties of workpieces. To address these shortcomings, models grounded in dislocation mechanics are devised in this paper, offering a more physical depiction of material plasticity. Although the numerical model presents computational challenges, it provides a more profound understanding of the intrinsic metallurgical processes that take place during manufacturing, with material behavior being contingent upon microstructural evolution.

This paper introduces a physics-informed model that encapsulates dislocation motion and density progression, simulating the plastic response of Inconel 718 to the substantial strain deformation characteristics of machining operations. Analogous simulation analyses were conducted to glean further insights into the microstructural evolution mechanisms, potentially enhancing the machinability of final products. The findings encompass variables of machinability, including forces and temperatures, offering an in-depth perspective on the phenomena inherent to the high-speed machining of Inconel 718 components.

## 2. Materials

The focus of this study is to develop and validate a program to simulate the formation process of chips, which can predict the influence of a workpiece’s microstructure on its machining performance. The analysis primarily focuses on Inconel 718, a material recognized for its superior resistance to fatigue, radiation, oxidation, and corrosion. The microstructure of Inconel 718 mainly consists of γ, γ′, γ″, and δ phases, MX-type carbonitrides, and Laves phases. Inconel 718 is primarily composed of Ni (approximately 55%), Cr (approximately 21%), and Fe (approximately 16%). The alloy’s superior mechanical properties are characterized by a high elastic modulus of 199.9 GPa, tensile strength of 965 MPa, and yield strength of 550 MPa. However, its relatively low thermal conductivity of 14.7 W/m·K and specific heat capacity of 435 J/kg·K can lead to excessive cutting temperatures during machining processes. The elemental composition of materials can typically be obtained using methods such as electron scattering, X-ray photoelectron spectroscopy (XPS), and elemental mapping [26,27]. Table 1 illustrates the crystallographic structure and composition of Inconel 718’s predominant phases. Its intricate precipitates, namely γ′, γ″, and δ, along with their diverse chemical compositions, significantly influence the alloy’s flow characteristics and mechanical behavior.

The γ′ phase, characterized by a globular morphology and an ordered face-centered cubic (FCC) L1_2_ crystal structure, is a key precipitate in nickel-based superalloys. These γ′ precipitates consist of a Ni3Al-based composition, along with an L1_2_ structure, where Al atoms occupy the corner sites and Ni atoms fill the face-centered positions. A distinguishing micro-mechanical feature of the Ni_3_Al phase is the super-dislocation mechanism, which contrasts with the conventional dislocation movement in FCC structures, possessing a Burgers vector approximately twice as large. With a rising temperature, dislocations within the γ′ phase, predominantly screws, are prone to locking into the Kear–Wilsdorf (KW) structure due to their interaction with transverse slip.

The γ″ phase is recognized as the primary strengthening agent in Inconel 718, presenting as a disk-shaped, metastable precipitate with an ordered body-centered tetragonal (BCT) D0_22_ crystal structure, preferentially aligned along the {001} planes. Despite the δ phase sharing a composition akin to the γ″ phase, it does not contribute substantially to the alloy’s strength. The δ phase, distinguished by its needle-like shape and orthorhombic D0_a_ crystal structure, represents the equilibrium state to which the γ″ phase corresponds.

## 3. Microstructure-Based Flow Stress Model Proposal

This section, potentially organized by subheadings, should deliver a succinct and accurate account of the experimental findings, an analysis of their implications, and the conclusions that can be inferred from these experiments.

The modeling of Inconel 718 requires not only considering small-scale microstructural mechanisms such as dislocation activity, but also establishing connections with higher-scale microstructures to simulate the microstructures of polycrystals. Attaining this objective necessitates the formulation of a microstructure-sensitive flow stress model, predicated on input parameters reflective of microstructural attributes. Acknowledging the constraints inherent to current constitutive models, this approach should address the following: the material’s response to elevated strains, strain rates, and temperatures, as well as the interplay among these factors.

The microstructure-based flow stress model is presented in Equation (1), with distinct components for strain hardening (*σ_SH_*), thermal softening (*σ_TS_*), and strain rate hardening (*σ_SRH_*). In addition, *σ_SH_* has stress units, and *σ_TS_* and *σ_SRH_* are dimensionless.

(1) $$\sigma =\sigma_{SH}\left(\varepsilon \right)\cdot \sigma_{TS}\left(T\right)\cdot \sigma_{SRH}\left(\dot{\varepsilon },T\right),$$

Only *σ_SH_* was obtained from the microstructure of Inconel 718, and *σ_TS_* and *σ_SRH_* are determined through Split-Hopkinson Pressure Bar (SHPB) testing.

### 3.1. Strain Hardening (σ_SH_)

*σ_SH_* follows a dislocation forest-hardening model. Strain hardening evolution adheres to a composite law involving macroscopic flow stress and strain increments. In plastic deformation, dislocation and its movement and interaction with microstructure play an important role. Plastic deformation results from the initiation and movement of dislocations within the crystal lattice. It is influenced by interactions with the material’s microstructure, including obstacles such as immobile dislocations, solutes, precipitates, and defects, which impede dislocation motion. Conventionally, the effects of these microstructural features on the macroscopic flow stress are considered to be additive, as illustrated in Equation (2), where the individual contributions of the lattice, dislocations, solutes, precipitates, and defects are aggregated.

(2) $$\sigma_{SH}=\sigma_{i}+\sigma_{HP}+\sigma_{G}+\sigma^{*},$$

where *σ_i_* represents the internal friction stress, also known as the Peierls–Nabarro stress, resulting from dislocation motion through an ideal lattice, *σ_HP_* corresponds to the grain-size-dependent stress attributed to the Hall–Petch effect, *σ_G_* denotes the athermal stress from long-range lattice disturbances by immobile dislocations, often referred to as forest dislocations, and σ^*^ signifies the stress associated with short-range interactions, required to displace dislocations past local obstacles.

In this model, the evolution of the flow stress structure is calculated based on the dislocation density and vacancy concentration as internal state variables, according to the principle of statistical thermodynamics. The vacancy concentration depends on the temperature, and a certain temperature corresponds to a certain equilibrium vacancy concentration. Vacancy can be formed in the process of material production, or it can be formed by other ions or electron implantation. The distortion of the crystal lattice caused by dislocation and other defects makes dislocation movement more difficult, that is, plastic deformation.

Additionally, other contributions, deemed insignificant for Inconel 718, are not accounted for in this model. The internal friction stress is omitted from the current model; instead, its influence is integrated into the initial dislocation density, which contributes to the long-range component defining the virgin yield limit. *σ_HP_* encompasses the stress concentration effects at grain boundaries and the supplementary stress needed for plastic deformation transfer across grain boundaries.

(3) $$\sigma_{HP}=\frac{k_{FP}}{\sqrt{g}},$$

where *k_FP_* is the calibration parameter and *g* is the grain size.

Flow stress is partitioned into the long-range contribution, *σ_G_*, and the short-range component, σ^*^, based on the movement of lattice dislocations. *σ_G_* is called non-thermal vibration, because thermal vibration cannot help dislocations to overcome the long-range disturbance of the lattice. *σ_G_* can be calculated using Equation (4) [28].

(4) $$\sigma_{G}=\alpha m\mathrm{Gb}\sqrt{\rho_{i}},$$

where *α* is a proportionality interaction factor (constants related to the crystal structure, usually in the range of 0.2–0.5), m is the mean Taylor factor, *G* is the temperature dependent shear modulus, *b* is the Burger’s vector, and *ρi* is the immobile dislocation density.

σ^*^ signifies the material’s resistance to plastic strain, where thermally activated processes complement the applied stress in facilitating dislocation movement through the crystal lattice. Commonly, dislocation and crystal defect interactions can manifest as short-range barriers that thermal activation helps to surmount. As dislocations traverse the lattice, they face obstacles, and the interval—termed the ‘flight time’—required to pass from one impediment to the next is considered to be minimal relative to the waiting time. The rate of successful dislocation jumps, indicative of overcoming these barriers, correlates with the likelihood of energy levels surpassing the requisite activation energy, expressible via an Arrhenius-type relationship. Consequently, the mean velocity of dislocations is delineated by the kinetic equation.

(5) $$\bar{v}=\beta b\nu_{a}e^{\frac{-\Delta G}{kT}},$$

where *β* is a dimensionless constant, *v_a_* is the attempt frequency related to the oscillations in the lattice, ∆*G* represents the activation energy, *k* is the Boltzmann constant, and *T* signifies the absolute temperature. The relationship between the plastic strain rate and dislocation velocity is captured by the Orowan equation [29].

(6) $$\dot{\bar{\varepsilon }}=\frac{\rho_{m}b\bar{v}}{m},$$

where *ρ_m_* signifies the density of mobile dislocations. Combining Equations (5) and (6):

(7) $${\dot{\bar{\varepsilon }}}^{p}=\frac{\rho_{m}\beta b^{2}\nu_{a}}{m}e^{\frac{-\Delta G}{kT}}=f\left(\sigma \right)e^{\frac{-\Delta G}{kT}}\approx {\dot{\bar{\varepsilon }}}_{ref}e^{\frac{-\Delta G}{kT}},$$

**Figure 1:**
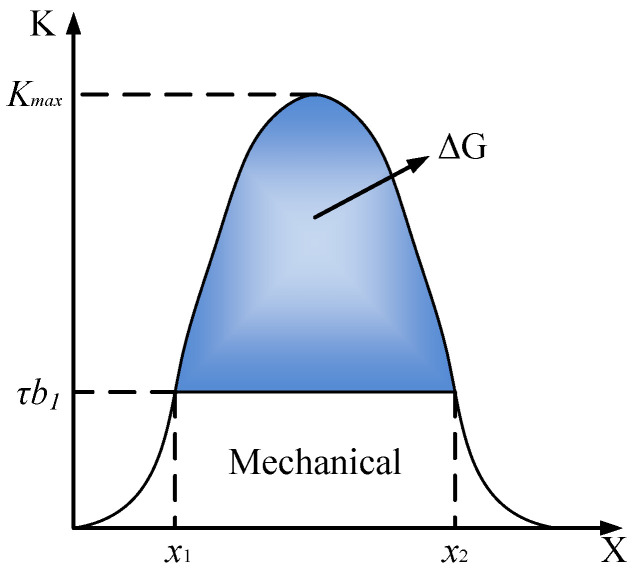
Variation in energy consumed by dislocation motion with respect to movement distance.

In the case of dislocation movement, the activation energy might be considered as the energy barrier that dislocations must overcome to move through the crystal lattice. This energy is necessary to break the bonds or interactions that hold the dislocations in place, allowing them to glide or climb to new positions. Dislocation motion during thermally activated glide is aided by thermal activation energy. If the applied stress is inadequate to propel a dislocation past an obstacle, thermal energy can provide supplementary assistance. The energy needed to surmount an obstacle is determined by the barrier’s height and shape. As depicted in Figure 1, *τb*_1_ represents the energy barrier, and K is the energy consumed by the dislocation motion. It can be observed from the diagram that, once the dislocation reaches the *x*_1_ position (at which point, it needs to overcome the energy barrier), additional energy (∆*G*) is required to surmount the barrier and proceed to *x*_2_. This requisite activation energy is calculated as the difference between the total energy and the mechanical energy contribution.

The Gibbs free energy correlates with both the distribution of obstacles and the barrier’s profile, which the dislocation must surmount. A general formula for the activation energy is [30]:

(8) $$\begin{array}{cc} \Delta G=\Delta F_{0}{\left[1-{\left(\frac{\sigma *}{\sigma_{ath}}\right)}^{p}\right]}^{q} & 0\leq p\leq 1,1\leq q\leq 2 \end{array},$$

where Δ*F*_0_ represents the free energy needed to overcome obstacles, *σ_ath_* denotes the athermal flow stress essential for bypassing these obstacles, while *p* and *q* serve as calibration coefficients.

**Figure 2:**
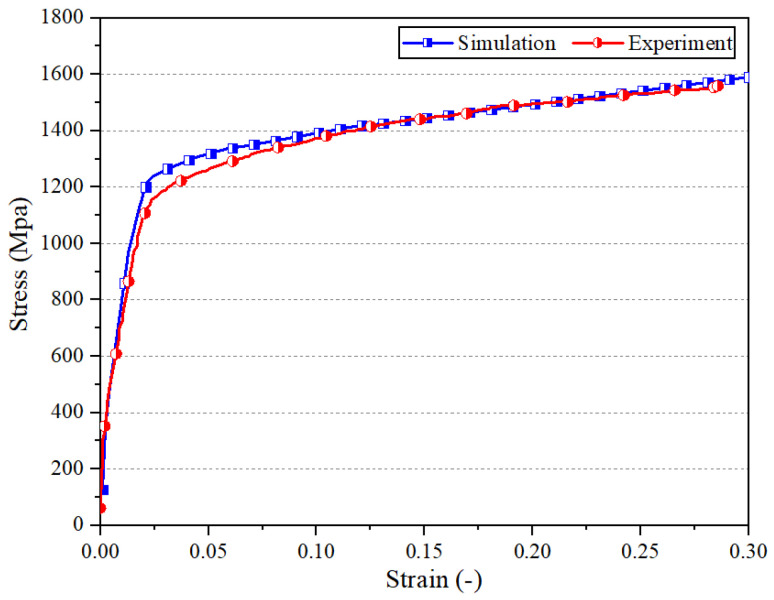
A comparison of flow stress curves, derived from both predictions and experiments at 20 °C with a strain rate of 0.5 s^−1^.

The free energy required to surmount obstacles is expressed as Δ*F*_0_ = Δ*f*_0_*Gb*^3^, and the athermal flow stress is given by *σ_ath_* = *τ*_0_*G*. Table 2 provides the activation energy and shear strength factors across various obstacles. The shear strength factor is a parameter used to describe a material’s ability to resist deformation under shear, and it is related to the material’s yield strength. It measures the maximum shear stress that a material can withstand before plastic deformation occurs. *τ*_0_ is a measure of obstacle strength, expressed as a dimensionless quantity, Δ*f*_0_ is a calibration coefficient, and *l* indicates the average obstacle spacing. The short-range stress component can be obtained by Equations (7) and (8). The constant parameters are shown in Table 3. Figure 2 illustrates the model’s accuracy in predicting the behavior of Inconel 718 under uniaxial compression at 20 °C and a strain rate of 0.5 s^−1^, with the results closely mirroring those from room temperature quasi-static compression tests.

(9) $$\sigma *=\tau_{0}G{\left[1-{\left[\frac{kT}{\Delta f_{0}Gb^{3}}\ln\left(\frac{{\dot{\bar{\varepsilon }}}_{ref}}{{\dot{\bar{\varepsilon }}}^{p}}\right)\right]}^{\frac{1}{q}}\right]}^{\frac{1}{p}},$$

where ${\dot{\bar{\varepsilon }}}_{ref}$ is the reference strain rate.

### 3.2. Thermal Softening (σ_TS_)

The effect of thermal softening on flow stress was investigated using the SHPB experiment. In this experiment, Inconel 718 was used as the specimen material. The test specimens were prepared by wire cutting from Inconel 718 rods. To manipulate the deformation strain rate across various conditions during testing, cylindrical Inconel 718 specimens of two sizes were machined along the rod’s axial direction. One set of specimens measured 3 mm in diameter and height, while the smaller set was 2 mm in both dimensions. During specimen preparation, the bottom and top surfaces were ground and polished to ensure precision in the testing and to reduce friction with the testing apparatus.

**Figure 3:**
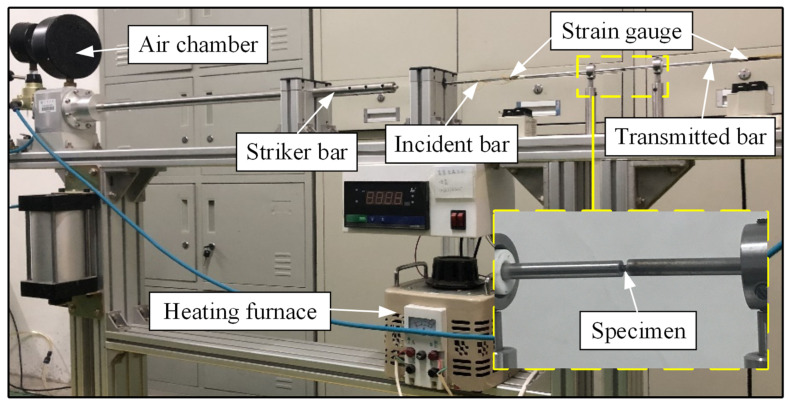
SHPB experimental equipment.

Figure 3 illustrates the schematic of the SHPB setup. Tests were performed at strain rates between 5000 s^−1^ and 11,000 s^−1^ over a temperature range from 20 °C to 800 °C. In the high-temperature trials, a resistance wire heated the specimen to a furnace-maintained temperature. The furnace temperature error range was maintained within ±5 °C using a closed-loop controller connected to a thermocouple. The specimens were heated to a fixed temperature by enclosing them in the furnace and holding them at that temperature for 15 min to ensure uniform heating before the impact test. Strain gauges affixed to the incident and transmission bars detected stress pulse variations. Amplified by a signal amplifier, these pulses yielded two sets of piezoelectric signal traces on the data acquisition system. Subsequently, the signals were analyzed using one-dimensional wave theory after processing.

**Figure 4:**
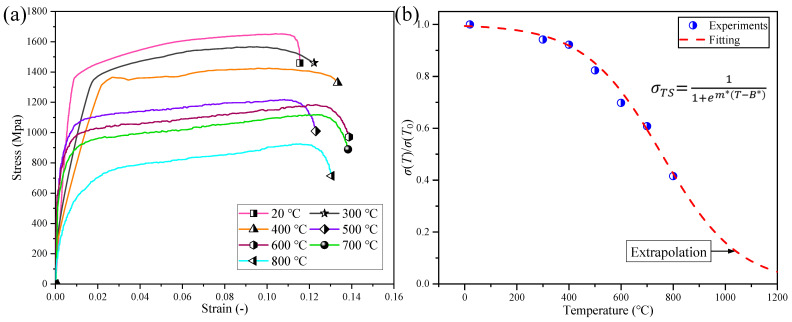
Thermal softening effect, (**a**) stress–strain curves of Inconel 718 obtained at different temperatures and (**b**) fitting of characterization formula for thermal softening effect.

The stress–strain curves of Inconel 718 were obtained through SHPB bar compression experiments with a strain rate of 5000 s^−1^ and temperature range of 20 °C–800 °C. The nonlinear thermal softening effect of Inconel 718 was then described using an exponential formula (Equation (10)) [32]. The flow stress at various temperatures relative to the value at *T*_0_ = 20 °C is shown in Figure 4b. The values of m* and B* obtained through curve fitting were 0.0068 °C^−1^ and 756 °C, respectively.

(10) $$\sigma_{TS}=\frac{1}{1+e^{m^{*}\left(T-B^{*}\right)}},$$

where m* and B* are the temperature sensitivity coefficients.

### 3.3. Strain Rate Hardening (σ_SRH_)

**Figure 5:**
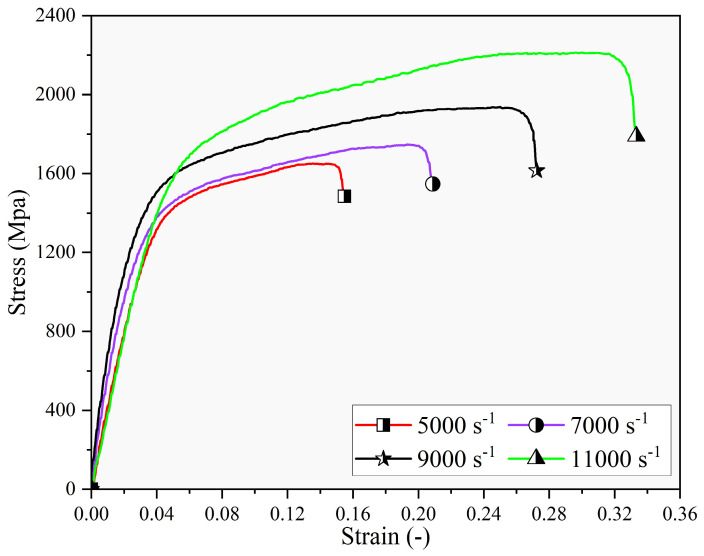
The impact of strain rates on the stress–strain curves.

To ascertain the strain rate hardening effect of the workpiece, the SHPB test was conducted to elucidate the influence of varying strain rates (5000, 7000, 9000, and 11,000 s^−1^) on the stress–strain curves of the materials at a constant temperature of 20 °C, as illustrated in Figure 5. The figure indicates a direct correlation between increased strain rates, ranging from 5000 to 11,000 s^−1^, and a heightened flow stress. The strain rate’s impact on Inconel 718 is captured by the strain rate hardening component of the JC model, as detailed by Equation (11). Parameter C of the strain rate hardening term in the constitutive model was determined to be 0.0133 by employing the method of linear regression.

(11) $$\sigma_{SRH}\left(\dot{\varepsilon }\right)=1+C\cdot ln\left(\frac{\dot{\varepsilon }}{{\dot{\varepsilon }}_{0}}\right)$$

where *C* is the constitutive model parameter in the strain rate hardening term.

## 4. Verification of Flow Stress Model Based on Microstructure

### 4.1. Cutting Experimental Design

**Figure 6:**
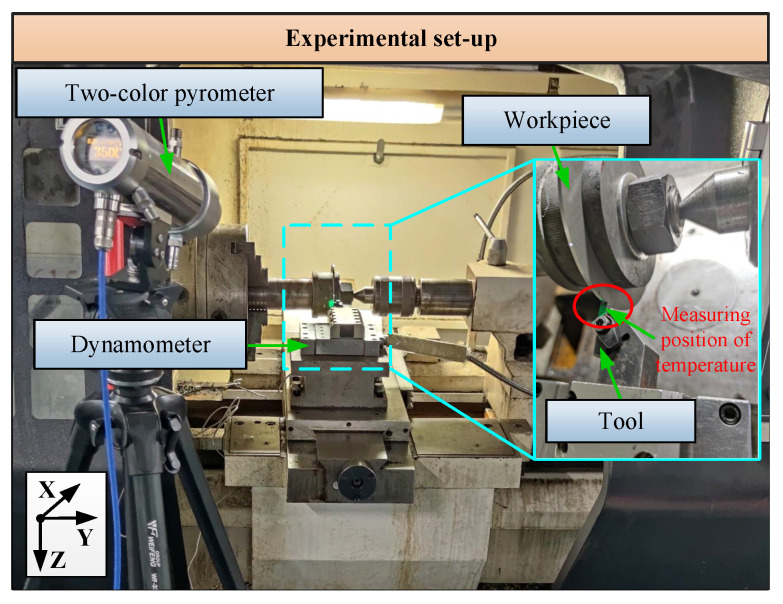
Cutting experiments’ setup.

The orthogonal cutting of Inconel 718 radial disks was performed using uncoated tungsten carbide inserts, with the experimental setup depicted in Figure 6. The experimental apparatus was based on a CNC lathe. The cutting parameters included various cutting speeds *v* = 30, 45, 60 m/min, an undeformed chip thickness *t*_0_ = 0.1 mm, and a cutting width *a*_*w*_ = 2  mm.

Cutting force and temperature are indicative of both the operational state and the inherent material processability, reflecting the complexity of the processing process. A Kistler 9257B piezoelectric dynamometer, positioned beneath the tool, was utilized to gauge the cutting forces. Concurrently, the chip’s cutting temperature was monitored in real time with a two-color pyrometer (STRONG-GR-3514), capturing temperatures spanning from 350 °C to 1400 °C. This pyrometer offers a measurement precision of ±0.1% of the true temperature (*tm*) with a granularity of 0.1 °C. Each set of measurements was conducted a minimum of five times to ensure reliability, with the mean and variability being subsequently determined.

### 4.2. Comparison of Cutting Forces

**Figure 7:**
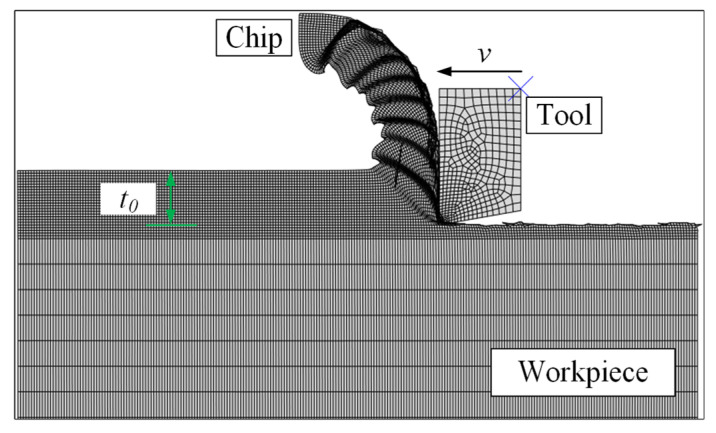
The mesh partitioning of the model and the simulation of the cutting process.

The constitutive model established through the VUMAT subroutine was integrated into the main program of Abaqus for further prediction of the cutting forces and cutting temperatures during orthogonal cutting under the same cutting parameters. Figure 7 illustrates the model’s mesh partitioning and the simulated cutting process, with the simulation outcomes compared against the experimental measurements.

**Figure 8:**
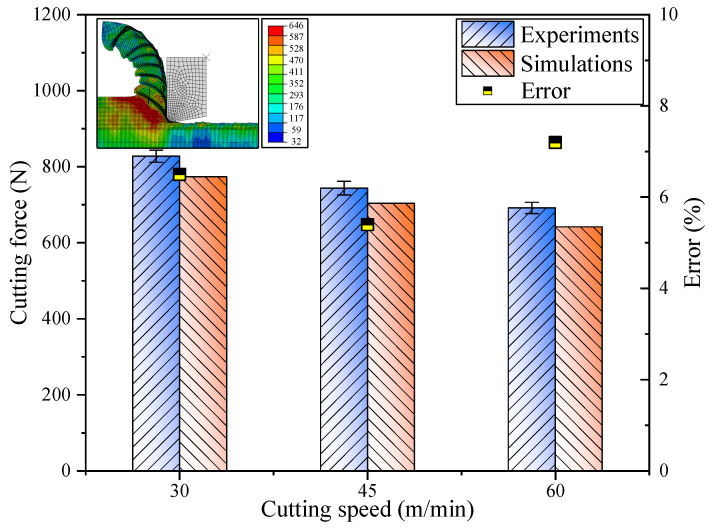
Comparison of experimentally measured cutting forces with simulated cutting forces.

Figure 8 illustrates a comparative analysis of the cutting forces (Fc) obtained from experimental measurements and those simulated through computational modeling. The experimentally determined cutting forces at varying cutting velocities were recorded at 828 N, 744 N, and 692 N, correspondingly. Conversely, the simulated cutting forces, as forecasted by the micro-flow stress model, were 774 N, 704 N, and 642 N, respectively. The discrepancies in the predicted cutting temperatures in relation to the experimental outcomes were quantified at 6.5%, 5.4%, and 7.2%, respectively, demonstrating a commendable congruence between the empirical data and the projected temperatures. The predictive values marginally deviated below the empirical values.

### 4.3. Comparison of Cutting Temperatures

**Figure 9:**
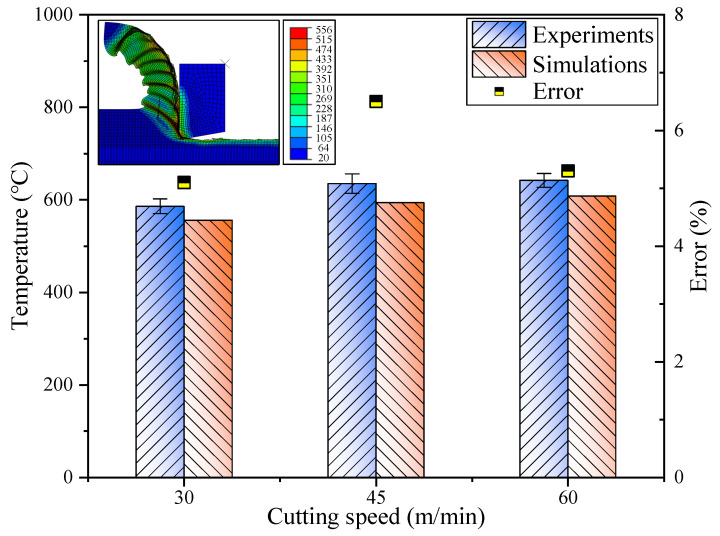
Comparison of experimentally measured cutting temperatures with simulated cutting temperatures.

The majority of the deformation and frictional energies generated during the cutting process were transformed into thermal energy. With the thermal influence of the cutting heat source, the temperatures of the chip, workpiece, and cutting tool escalated. The phenomena of tool wear and built-up edge were markedly influenced by the cutting temperature. Elevated cutting temperatures may also result in a compromised surface integrity, such as increased tensile residual stresses and the presence of white layers on the machined surface. Consequently, the accurate forecasting of cutting temperatures is of paramount importance when machining Inconel 718. Figure 9 juxtaposes the experimentally measured and simulated cutting temperatures. The experimentally determined cutting temperatures at various cutting speeds (30, 45, and 60 m/min) were 586 °C, 635 °C, and 642 °C, respectively. Meanwhile, the simulated cutting temperatures predicted by the microstructure-based flow stress model were 556 °C, 594 °C, and 608 °C, respectively. The relative differences in the predicted cutting temperatures versus the experimental data at varying cutting speeds were 5.1%, 6.5%, and 5.3%, respectively. The underestimation of temperatures in the predictions can predominantly be attributed to the omission of the effects of tool wear in the simulation forecasts.

## 5. Conclusions

This paper introduces a microstructure-informed constitutive model for Inconel 718, developed through an analysis of dislocation dynamics and density changes, as well as SHPB testing. This model quantitatively forecasts the alloy’s machinability with respect to cutting forces and temperatures, correlating these predictions with outcomes from orthogonal cutting studies. Overall, the trends observed in the simulations aligned well with the outcomes obtained from the orthogonal cutting experiments. Scientific variables such as cutting forces and cutting temperatures were predicted with an error margin ranging between 5 and 8%.

## Figures and Tables

**Table 1:** Crystal structure and composition of main phase of Inconel 718.

Phase	Formula	Crystal Structure	Lattice Constant/nm
γ	-	FCC (Al)	a = 0.3616
γ′	Ni_3_(Al, Ti)	FCC (L1_2_)	a = 0.3589
γ″	Ni_3_Nb	BCT (D0_22_)	c = 0.7406(c/a = 2.04)
δ	Ni3Nb	Orthogonal (D0_a_)	a = 0.5141, b = 0.4231, c = 0.4534
MX	(Nb, Ti)(C, N)	FCC (B_1_)	a = 0.443~0.444
Laves	(Ni, Cr, Fe)_2_(Nb, Mo, Ti)	Hexagonal	-

**Table 2:** Activation energy and shear strength factors across various obstacles.

Obstacle Strength	Δ*f*_0_	*τ*_0_	Example
Strong	2	$>\frac{b}{l}$	Strong precipitates
Medium	0.2–1.0	$\approx \frac{b}{l}$	Weak precipitates
Weak	<0.2	<$\frac{b}{l}$	Lattice precipitates

**Table 3:** Predefined or estimated parameters [31].

Parameter	Meaning	Value	Unit
α	A proportionality interaction factor	0.35	-
*k*	Boltzmann constant	1.38 × 10^−23^	J/K
m	The mean Taylor factor	3.06	-
*b*	Burger’s vector	2.54 × 10^−10^	m
${\dot{\bar{\varepsilon }}}_{ref}$	Reference strain rate	1 × 10^6^	s^−1^
*g*	Grain size	20 × 10^−6^	m
*G*	The temperature dependent shear modulus	142.2	GPa
*p*	Calibration constants	1	-
*q*	Calibration constants	1.5	-
$\rho_{i}$	The immobile dislocation density	5.0 × 10^15^	m/m^3^
$\Delta f_{0}$	Calibration constant	1	-
$\tau_{0}$	Dependent on the obstacle’s strength	5.62 × 10^−3^	-
*k_FP_*	Calibration parameter	34	-

## Data Availability

Data are contained within the article.
